# Robotic assistance during cochlear implantation: the rationale for consistent, controlled speed of electrode array insertion

**DOI:** 10.3389/fneur.2024.1335994

**Published:** 2024-01-22

**Authors:** Rustin G. Kashani, Allan Henslee, Rick F. Nelson, Marlan R. Hansen

**Affiliations:** ^1^Department of Otolaryngology – Head and Neck Surgery, University of Iowa Hospitals and Clinics, Iowa City, IA, United States; ^2^iotaMotion, Inc., Iowa City, IA, United States; ^3^Indiana University, Indianapolis, IN, United States

**Keywords:** cochlear implantation, hearing preservation cochlear implantation, hearing preservation, robotic surgery, robotic cochlear implantation, insertion tool, insertion trauma, electrode array

## Abstract

Cochlear implants (CI) have revolutionized the treatment of patients with severe to profound sensory hearing loss by providing a method of bypassing normal hearing to directly stimulate the auditory nerve. A further advance in the field has been the introduction of “hearing preservation” surgery, whereby the CI electrode array (EA) is carefully inserted to spare damage to the delicate anatomy and function of the cochlea. Preserving residual function of the inner ear allows patients to receive maximal benefit from the CI and to combine CI electric stimulation with acoustic hearing, offering improved postoperative speech, hearing, and quality of life outcomes. However, under the current paradigm of implant surgery, where EAs are inserted by hand, the cochlea cannot be reliably spared from damage. Robotics-assisted EA insertion is an emerging technology that may overcome fundamental human kinetic limitations that prevent consistency in achieving steady and slow EA insertion. This review begins by describing the relationship between EA insertion speed and generation of intracochlear forces and pressures. The various mechanisms by which these intracochlear forces can damage the cochlea and lead to worsened postoperative outcomes are discussed. The constraints of manual insertion technique are compared to robotics-assisted methods, followed by an overview of the current and future state of robotics-assisted EA insertion.

## Introduction

Cochlear implants (CI) have quickly become the predominant intervention for treating patients with severe to profound sensory hearing loss. Historically, CIs were used mostly in patients who had minimal to no remaining acoustic hearing, as it was thought that introduction of the electrode array (EA) into the cochlea would compromise any residual structure and function. Since the 1980s, further investigation has found that preservation of intracochlear anatomy and residual neural and sensory function including existing acoustic hearing was an achievable and desirable goal for CI recipients (1). This functional preservation allowed the combination of CI electric stimulation with residual acoustic hearing, also called “electroacoustic stimulation” (EAS), initially described by von Ilberg et al. (2). The first clinical trial of 6 millimeter (mm) and 10 mm electrodes demonstrated the ability to preserve residual acoustic hearing (3). Compared to patients with electric stimulation alone, EAS offers improved speech understanding in noise, better sound localization, appreciation of music, and improved quality of life (4, 5). These findings provide the basis for “hearing preservation” surgery, whereby surgeons attempt to limit trauma to the delicate intracochlear tissues during insertion of the EA. Even in patients with non-functional acoustic hearing, EA insertion trauma is associated with decreased neural survival and poorer patient performance (6, 7). Yet despite best efforts, the fine structures of the cochlea cannot be reliably spared during implantation – at least under the current surgical paradigm. To date, a number of factors are known to contribute to the success of structural and functional preservation CI surgery, including the EA design (8), medications such as steroid delivery (9), surgical approach (10), and the technique as well as speed of EA insertion (11–14). The vast majority of EAs are inserted by hand, termed “manual insertion,” but this method suffers from the inherent limitations of human kinetics.

Robotics-assisted EA insertion is an emerging technique in structure and function preservation CI surgery that has the potential to overcome the limitations of a human operator. The scientific evidence supporting the use of robotic assistance during EA insertion is well-established and continues to quickly expand. The purpose of this mini-review is to explore the current understandings of the relationship between consistent, controlled insertion speed, and cochlear trauma, describe the current state of robotics-assistance EA insertion platforms, and discuss areas for future development. Below, a brief summary of the literature investigating the utilization of robotics-assistance in CI surgery is presented. Specifically, the current body of literature supports the following statements:

1. A slow and consistent EA insertion speed reduces insertion forces and intracochlear pressure spikes
2. Reduced insertion force limits intracochlear trauma and preserves structural integrity
3. Reduced trauma may improve hearing outcomes

## A slow and consistent EA insertion speed reduces insertion forces and intracochlear pressure spikes

EA insertion imparts a variety of intracochlear forces that act via different mechanisms. First, direct contact from the EA with cochlear structures delivers forces along the lateral wall tissues, basilar membrane, and osseous spiral lamina. These forces can increase if the electrode kinks or buckles as it meets resistance (15). Resistance to EA insertion typically begins at an insertion depth of 5 mm into the cochlea, then may spike at 8–10 mm when the electrode tip encounters the basal turn or back wall of the cochlea (16). Second, EA insertion also produces frictional forces arising from the interaction between the EA and the endosteum lining, with perilymph serving as a lubricant (17). Frictional forces are thought to increase as the area of contact between the array and lateral wall increases, which may explain why insertion forces tend to continue rising with deeper insertion (18). The relationship between insertion speed and friction may not be linear. Dohr et al. determined that the friction coefficient in a synthetic model was lowest at an insertion speed of 0.01 mm/s, highest at a speed of 1.5 mm/s, and then dropped again at a speed of 2 mm/s (17). A study done by Miroir et al. found no relationship between speed and friction forces (18). Third, the EA occupies space in the fluid filled compartment of scala tympani, which creates hydraulic pressure due to displacement of perilymph. Such increases in intracochlear pressure, often seen as extreme spikes during manual insertion, mirror exposure to excessive noise (13, 19). Slow and steady EA insertion, such as enabled by robotics assistance, has been linked to reduced hydraulic force and spikes in intracochlear pressure (19) by facilitating gradual perilymph egress/pressure equalization as the EA displaces more fluid (20), though there are reports that have not replicated this association (21).

**Figure 1:**
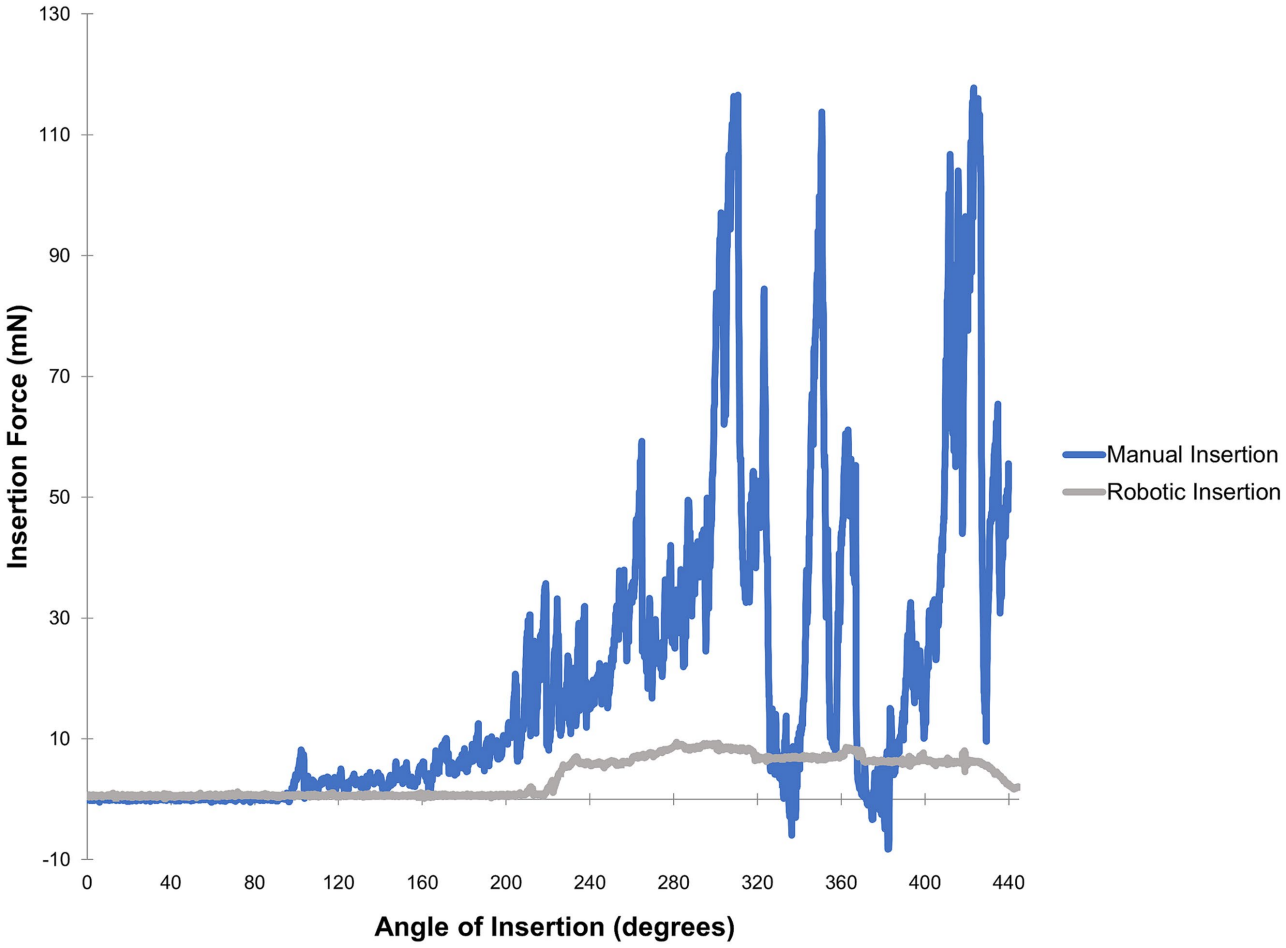
Force profiles obtained from a trial of manual versus robotics-assisted EA insertion. A polytetrafluoroethylene phantom model fabricated as described by Clark et al. (30) was used along with a MED-EL FLEX24 electrode array and 10% solution dish soap in distilled water for lubrication. A neurotology attending was instructed to perform insertions as slowly as possible, first via manual insertion then followed by robotics-assisted insertion with the iotaSOFT system. A 6-axis force sensor was used for measurements. These force profiles are representative of data comparing automated versus manual insertions (11, 28, 31).

When considering overall intracochlear force, studies have generally found that the speed of EA insertion is directly and positively correlated with generation of intracochlear force. In a synthetic cochlea model, Kontorinis et al. demonstrated that progressive increases in insertion speed resulted in greater average and maximum insertion force, with the lowest insertion force correlating with the lowest speed tested of ~0.16 millimeters per second (mm/s) (14). For comparison, the average speed of EA insertion for cochlear implant surgeons is 10 times higher at approximately 1.6 mm/s (14). Other studies using synthetic models corroborated this general trend using various EAs, speeds, and insertion techniques; particularly when evaluating peak forces (11, 22, 23). In cadaveric bones, higher insertion forces are correlated with higher rates of trauma, especially as the EA traverses the basal turn of the cochlea (24, 25). It has also been demonstrated that an insertion with robotic control significantly reduces the force variability or “jerkiness” of insertion compared to manual insertion (26). Additionally, insertion of an EA is associated with increased intracochlear pressure transients, akin to high levels of noise exposure. As noted above, pressure transients can be comparable to ear canal sound pressure levels of approximately 134–174 dB sound pressure level, similar to blast explosions associated with hair cell loss (13). These intracochlear pressure transients are related to the speed of insertion and robotic assistance significantly decreases these potentially deleterious events (27). By slowing insertion speed, the amplitude and frequency of intracochlear pressure spikes can be lessened (13, 20, 28, 29). Figure 1 illustrates representative differences in intracochlear force generation between manual and robotics-assisted EA insertion performed using an experimental phantom cochlea model.

## Reduced insertion speed and forces limit intracochlear trauma

Very small forces applied to the cochlea can disrupt inner ear structure and function. As little as 42 millinewtons (mN) can damage the cochlea, with even lower thresholds for injury to the basilar membrane (26–35 mN) and Reissner’s membrane (4.2 mN) (32). Studies of intracochlear forces generated during manual insertion show wide variability with reported forces varying from 0.310 to 0.420 newtons (N), as summarized in Table 1. Trauma from the forces incurred by EA insertion is thought to be a major source of cochlear injury. A review by Bas et al. outlines the primary mechanisms in which the insertion of the EA can lead to loss of auditory function including (45):

1. Direct injury to hair cells along the basilar membrane;
2. Inflammatory response including fibrosis;
3. Trauma to the spiral ligament and stria vascularis leading to disrupted endocochlear potential;
4. Unintentional rupture of the basilar membrane resulting in scalar translocation of the EA; and
5. Large pressure spikes that damage sensory cells in a fashion similar to acoustic injuries.

**Table 1:** Studies of cochlear implant insertion speed and maximum force or pressure.

Roland (2005) (33)	Nucleus contour advance	Temporal bone	Automated, SIT Automated, AOS	2.0 mm/s, 0.175 N 2.0 mm/s, 0.02 N
Todd (2007) (34)	Nucleus contour advance	Scala tympani model	Automated, SIT Automated, AOS	2.0 mm/s, 0.194 N 2.0 mm/s, 0.05 N
Radeloff (2009) (35)	Uncoated custom electrode Coated custom electrode	Temporal bone	Automated, SIT Automated, SIT	0.5 mm/s, 0.329 ± 0.077 N 0.5 mm/s, 0.172 ± 0.047 N
Rau (2010) (36)	Nucleus contour advance	Scala tympani model	Automated, AOS	0.5 mm/s, 0.04 N
Majdani (2010) (11)	Nucleus contour advance	Scala tympani model	Manual, AOS Automated, AOS	0.3 mm/s, 0.031 N 0.3 mm/s, 0.036 N
Kontorinis (2011) (14)	Nucleus contour advance	Scala tympani model	Manual, SIT	0.17 mm/s, 0.18 ± 0.003 N 1.33 mm/s, 0.32 ± 0.004 N 3.33 mm/s, 0.42 ± 0.008 N
Kobler (2015) (37)	MED-EL FLEX 20 MED-EL FLEX 24 MED-EL FLEX 28	Scala tympani model	Automated, SIT	0.5 mm/s, 0.062 N 0.5 mm/s, 0.040 N 0.5 mm/s, 0.070 N
Avci (2017) (38)	Custom electrode	Temporal Bone	Automated, SIT	0.5 mm/s, 0.0415–0.0530 N
Mittmann (2017) (39)	Nucleus contour advance Nucleus slim straight	Scala tympani model	Automated, SIT	0.48 mm/s, 1.12 ± 0.15 mm Hg 0.48 mm/s, 0.86 ± 0.05 mm Hg
Todt (2017) (40)	MED-EL FLEX 20	Scala tympani model	Manual, SIT	0.8 mm/s, 0.25 N
Hugl (2018) (23)	Custom electrode	Scala tympani model	Automated, SIT	0.03 mm/s, 0.0127 ± 0.0027 N 0.4 mm/s, 0.0187 ± 0.0022 N 2.0 mm/s, 0.0187 ± 0.0048 N
Kaufmann (2020) (26)	Various	Temporal bone	Manual, SIT Automated, SIT	0.1 mm/s, 0.085 N 0.5 mm/s, 0.076 N 1 mm/s, 0.072 N 0.1 mm/s, 0.054 N 0.5 mm/s, 0.060 N 1 mm/s, 0.058 N
	Various	Scala tympani model	Manual, SIT Automated, SIT	0.1 mm/s, 0.115 N 0.5 mm/s, 0.062 N 1 mm/s, 0.075 N 0.1 mm/s, 0.060 N 0.5 mm/s, 0.036 N 1 mm/s, 0.045 N
Rau (2020) (41)	MED-EL FLEX electrodes	Scala tympani model	Automated, SIT	0.03 mm/s, 0.060 N 0.4 mm/s, 0.107 N
Dhanasingh (2021) (42)	MED-EL FLEX 28	Scala tympani model	Automated, SIT	0.1 mm/s, 0.035 N 0.5 mm/s, 0.017 N 1 mm/s, 0.031 N 2 mm/s, 0.035 N 4 mm/s, 0.033 N
Zuniga (2021) (43)	MED-EL FLEX electrodes	Scala tympani model	Automated, SIT	0.03 mm/s, 0.026 N 0.11 mm/s, 0.044 N 0.4 mm/s, 0.066 N 0.9 mm/s, 0.065 N 1.6 mm/s, 0.110 N
Aebischer (2022) (44)	Custom	Scala tympani model	Automated, SIT	0.33 m/s, 0.044 ± 0.004 N
Zagabathuni (2023) (29)	MED-EL FLEX 28	Scala tympani model	Automated, SIT	0.15 mm/s, 133 Pa 0.3 mm/s, 137 Pa 0.6 mm/s, 144 Pa 1.2 mm/s, 402 Pa

SIT, standard insertion technique; AOS, advance off stylet; N, newtons; Pa, pascals; mm, millimeters; s, second.

For these types of trauma, expression of proinflammatory molecules such as TNFα and IL-1β can be triggered within the cochlea, leading to a cytokine cascade mediating apoptosis in hair cells (46–48). Similarly, large spikes in intracochlear pressure mimic high-intensity noise exposure and lead to the accumulation of free radicals and oxidative stress in the cochlea (24). Next, any trauma that causes mixing of the perilymph and endolymph (such as scalar translocation) or a disruption of the endocochlear potentials (such as damage to the stria vascularis), can also result in loss of cochlear function. Finally, intracochlear trauma and inflammation triggers fibrosis in the cochlea that has been associated with loss of residual hearing and diminished CI performance (7, 49, 50).

In addition to reducing the direct damage caused as the EA contacts intracochlear structures, slow EA insertion also reduces hydraulic force by facilitating gradual perilymph egress/pressure equalization while the EA enters the confined fluid-filled intracochlear space (20). Taken together, there are a variety of factors by which insertion trauma can lead to loss of residual sensory and neural function in the cochlea. As this trauma has been linked to poor postoperative outcomes following CI, the need for slow and steady insertion cannot be overstated.

## Reduced insertion trauma may improve hearing outcomes

Patients with residual acoustic hearing comprise up to 80% of current CI recipients and this will likely rise further in the future (51). The clinical research connecting intracochlear trauma to CI outcomes is extensive and primarily based on the improved postoperative outcomes observed among patients with preserved residual acoustic hearing. In these patients, combining both an acoustic stimulus (with a hearing aid) and electrical stimulus (via the CI) provides enhanced benefits versus electrical stimulation alone with respect to pitch perception (52), speech perception (3, 53, 54), noisy environments (55, 56), and music appreciation (57, 58). However, immediately post-operatively or even years following surgery, up to 50% of these patients will lose their residual hearing and associated benefits (59–62).

There is some evidence to suggest that slower insertion may improve postoperative clinical measures. Rajan et al. performed comparisons between patients implanted with a target “slow” insertion speed of 0.25 mm/s vs. a “fast” speed of 1 mm/s with the same EA (12). They found that patients undergoing slow insertion had significantly higher rates of postoperative hearing preservation, more complete EA insertions, and a decreased incidence of vestibular symptoms in a 24-hours period after implantation. Further studies are needed to confirm whether slower insertion speed consistently improves hearing outcomes and quality of life for patients.

Although typical conversations concerning intracochlear trauma revolve around residual hearing preservation, patients with little to no residual hearing significantly benefit from atraumatic insertions. The mechanisms here are also multifactorial but likely involve damage and degeneration of tissues necessary for faithful transmission of electrical stimulation such as spiral ganglion neurons (63, 64). Additionally, inflammatory responses produced by traumatic insertions can produce fibrosis and scar tissue surrounding the EA, which has been shown to increase impedance of the electrode and distance from the modiolus, thus limiting the effectiveness of the CI itself (65). The impact of fibrosis has also been shown to negatively impact consonant-nucleus-consonant word recognition scores, one of the major standard assessments of speech outcomes (66).

## The significance of consistent, controlled speed

Considering the body of evidence, it is clear why most surgeons attempt to perform slow and consistent insertions by hand. Despite these best efforts, manual techniques cannot reliably achieve these slow and steady EA insertions due to the limitations of human kinetics. A study by Kesler et al. described that the lower limit of a constant forward motion manual EA insertion lies at an average speed of 0.87 mm/s and noted that a 0.25 mm/s insertion rate is not feasible for human operators to achieve, supporting the need and clinical utility of robotic assistance to spare trauma to the cochlea (67). Comparisons of robotics-assisted insertion with manual insertion have demonstrated that robotics-assisted insertion is associated with reduced intracochlear force generation (11) and rates of trauma, including tip fold-over (68).

It is important to note that not every study of insertion has found an inverse relationship between speed and forces/trauma to the cochlea. Avci and colleagues found no difference in insertion forces when inserting electrodes into an artificial model between speeds of 0.05 and 2 mm/s (38). Dhanasingh et al. did not find a perfectly linear relationship between insertion speeds and forces, sometimes finding that relatively lower speeds produced greater forces. However, their overall data showed a trend towards the lowest forces at the slowest speeds, and maximal forces at the highest speeds (42). Kaufmann and colleagues found that at the slowest tested insertion speed of 0.01 mm/s, significantly higher insertion maximal insertion forces were measured during both robotic and manual insertions in a synthetic cochlea model but not in the cadaveric specimen (26). While the majority of studies suggest that insertion speed and forces are linearly related, this may not hold true at very slow speeds. An inflection point may exist where very slow insertion speeds could generate higher intracochlear forces and trauma. The mechanism may be that very slow speeds would allow the EA to make more contact with intracochlear structures and push out fluids that form a lubricious intervening layer between the array and the cochlea, thereby increasing friction/shear forces. Similarly, if there are instances where the EA momentarily ceases forward movement during insertion, static friction forces would be applied perhaps leading to more transient pressure spikes. Further study is needed to further clarify the relationship between ultra-slow speeds, steady forward motion, and force generation, as well as determining the optimal speed for insertion of a given EA.

Varied experimental conditions must be considered when assessing studies of insertion forces. Many investigations use solely phantom cochlear models made of acrylic or other synthetic materials. The composition of a given model will have a certain coefficient of friction, potentially influencing force measurements. Fresh frozen cadaveric temporal bone specimens more closely replicate *in vivo* cochlear anatomy and can be expected to have a different insertion force profile compared to models, which may be due to the presence and elasticity of the basilar membrane, among other characteristics. Insertion forces may be twice as high in synthetic models compared to cadaveric specimens (25). It is possible that reproducibility of results obtained from cadaveric specimens may vary given the natural variation in cochlear and intracochlear structures/dimensions between bones (69). Studies also diverge in how forces are measured, e.g., multi-axis versus single-axis measurement, the location of the force sensors, and use of open-channel models (69). Finally, studies have used a variety of speeds, electrode types, insertion techniques (e.g., standard versus advance-off stylet), and lubricating fluids.

## Current state of robotics-assistance platforms and future directions

In current practice, there are two major robotics-assistance platforms used for EA insertion. The RobOtol system (Collin, France) is an operative platform developed to work in restricted areas through the external ear. It has a dedicated microinstrument holder that can be used to insert an electrode via translational and rotational movements, at speeds as low as 0.1 mm/s (70). A clinical study using RobOtol for EA insertion found no difference in hearing outcomes between robotics-assisted and manual insertion in sequentially implanted patients (71). However it is important to note that this was a small study of <10 patients and was acknowledged to be underpowered to detect significant differences. Thus, larger studies are needed to evaluate the effectiveness of robotic-assisted cochlear implantation on patient outcomes.

The iotaSOFT Insertion System (iotaMotion Inc., Iowa City, IA) consists of a single-use sterile drive unit with a robotic drive motor that can also insert arrays as slowly as 0.1 mm/s (72). The current iotaSOFT system is compatible with lateral wall electrodes; its next iteration is expected be compatible with both lateral wall and perimodiolar electrodes. The RobOtol system can be used for both lateral wall and perimodiolar EA insertion (73). Both the iotaSOFT and RobOtol platforms are increasingly being used for implantation and ongoing research studies. Various other prototype automated insertion devices have been described (74, 75) including Cochlea Hydro Drive, a hydraulic tool using an infusion pump described by Rau which can insert arrays as slowly as 0.03 mm/s (41).

Both RobOtol and iotaSOFT are increasingly being used for implantation in the clinical setting and are also the subject of ongoing research. It is not clear yet if robotics-assisted EA insertion leads to improve postoperative outcomes; clinical work by Maheo and colleagues found no difference in postoperative speech outcomes after sequential implantation via manual insertion versus use of the RobOtol platform in a small subset of patients (71). Additional comparative clinical studies are needed to demonstrate the extent to which robotics-assisted EA insertion affords improves patient outcomes, as well as the cost-effectiveness of these technologies. Use of robotics systems will presumably impact costs relating to operating room usage (76). A study comparing manual insertion with robotics-assistance using the Robotol system reported a mean preparation time of 630 ± 301 s (71). However, duration of surgery did not significantly differ between the manual and robotic insertion groups. Preparation of the Iotasoft system in a study of robotics-assisted implantation required a mean duration of 55.8 s (72). Mean insertion time was 315 s. Over time, cost per procedure using a given robotic platform will likely decrease due to more efficient preparation and use in the operating room.

There are several areas for further development of robotics-assisted EA insertion platforms. While slower speed may reduce insertion forces, there still exists a need to elicit feedback from the cochlea to prevent injury. Incorporation of force measurement sensors into the robotic platform or affixed to the patient is feasible (77). Intraoperative electrocochleography (ECochG) is an additional area of investigation assessing whether changes in ECochG amplitude reflect or precede injury to the cochlea. Robotics-assisted EA insertion may facilitate more consistent ECochG responses, as the inability to manually hold the EA steady confounds the ECochG waveform (78, 79). Additionally, integration of ECochG responses or force measurements with a robotics-platform opens the possibility of automatically halting insertion based on feedback variables, avoiding inherent time lag required for a human response to stimuli. Combining robotics-assisted EA insertion with navigation software that can optimally orient the EA for insertion into the round window would be a strong adjunct, as insertion trajectory is considered a significant variable in force generation (34, 80, 81). Precise assessment of insertional depth based on tonotopic estimates is another area of development.

## Conclusion

As the candidacy for cochlear implantation expands, patients with significant residual sensory and neural function in the cochlea will undergo surgery. This underscores the need to preserve inner ear function and maximize postoperative outcomes, a goal that is not reliably achieved with manual insertion. Robotics-assisted EA insertion, coupled with feedback mechanisms such as force sensors, ECochG and image-guidance, represents a dynamic field that holds great promise for the future of cochlear implantation.

## Author contributions

RK: Writing – original draft, Writing – review & editing. AH: Conceptualization, Investigation, Writing – original draft, Writing – review & editing. RN: Investigation, Writing – review & editing. MH: Conceptualization, Investigation, Supervision, Writing – review & editing.
